# 

**Published:** 1905-02

**Authors:** 


*Sixtieth Year.*

VOL. LX.
WHOLE NUMBER,
DCLXXXXIX

FEBRUARY, 1905.

New Series.

VOL. XLIV

No. 7.

INDEXED
S. G. O.

### BUFFALO MEDICAL JOURNAL

Established 1845 by AUSTIN FLINT, M. D.

[illegible] OFFICE
JAN 30 1905

EDITOR:

WILLIAM WARREN POTTER, M. D.

ASSISTANT EDITORS:

WILLIAM C. KRAUSS, M. D. NELSON W. WILSON, M. D.

ASSOCIATE EDITORS:

JAMES WRIGHT PUTNAM, M. D. ERNEST WENDE, M. D.

JOHN PARMENTER, M. D. JOHN A. MILLER, PH. D.

HARVEY R. GAYLORD, M. D. MAUD J. FRYE, M. D.

